# Risk of antimicrobial resistance spreading via food loss and waste

**DOI:** 10.1186/s40249-025-01405-6

**Published:** 2026-02-06

**Authors:** Fanette Fontaine, Jorge Pinto Ferreira, Antonio Valcarce, Emmanuel Kabali, Junxia Song

**Affiliations:** 1 Food and Agriculture Organization of United Nations, Rome, Italy; 2 Department for Environment, Food and Rural Affairs, London, UK

**Keywords:** Antimicrobial resistance genes, Food waste management processes, Composting, Anaerobic fermentation, Circular economy

## Abstract

**Background:**

While the agricultural sector is a known contributor to antimicrobial resistance (AMR), the potential role of food loss and waste (FLW) in AMR dissemination has been largely overlooked. FLW, a byproduct of inefficient food systems, poses economic, environmental, and food security challenges. It may also act as a reservoir and vector for antimicrobial resistance genes and antibiotic-resistant bacteria, contributing to the environmental spread of AMR if improperly managed. This narrative review assessed the presence, fate, and risks of AMR in FLW management.

**Methods:**

Peer-reviewed studies were identified through systematic searches in PubMed and Web of Science using keywords related to food waste, AMR, and treatment methods. Additional studies were retrieved through reference screening. Only English-language articles addressing AMR in the context of FLW were included.

**Results:**

Bioconversion processes such as composting, anaerobic digestion, and conversion to animal feed can reduce antimicrobial resistance genes and antibiotic-resistant bacteria under optimized conditions. However, without adequate treatment, end products like fertilizers or biomaterials may still pose AMR risks. In contrast, FLW disposal in landfills and open dumps exacerbates both greenhouse gas emissions and AMR risks, due to co-contamination with other AMR-promoting pollutants like heavy metals and microplastics. AMR can spread through multiple pathways, including leachate, aerosols, wildlife, and direct human contact.

**Conclusions:**

FLW should be recognized as a potential AMR source, requiring improved management strategies and integration into AMR surveillance. This review highlights the need to both reduce antimicrobial use and minimize FLW generation to limit environmental and public health risks.

**Graphical Abstract:**

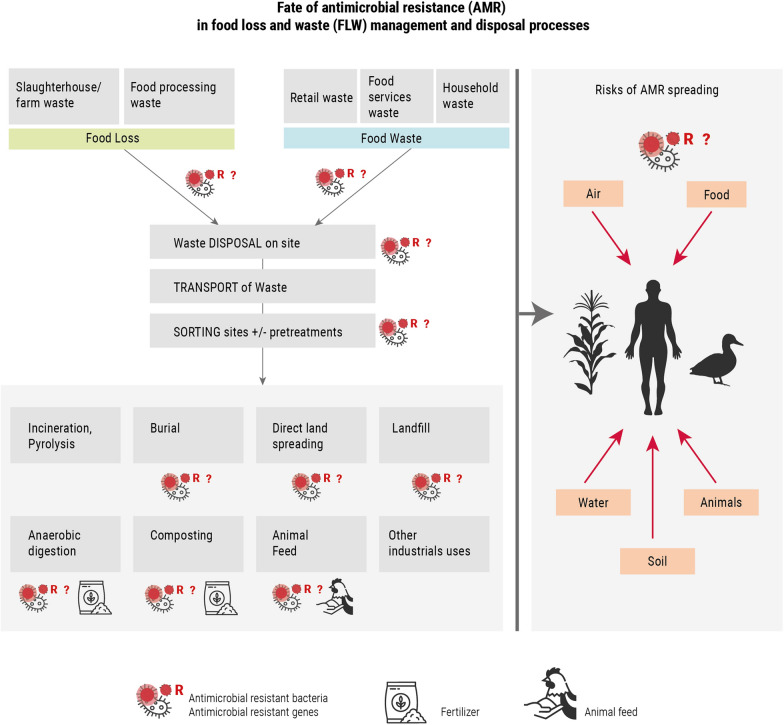

## Background

Antimicrobial resistance (AMR) poses a significant global threat to public health, animal health, and environmental sustainability. Agriculture is both a major user of antimicrobials and a key contributor to AMR [1, 2]. Antimicrobials are predominantly used in animal production—accounting for about 73% of global antibiotic global sales—for a variety of purposes beyond disease treatment, including disease control and prevention, and as feed proficiency enhancers (i.e., growth promoters) [3]. To a lesser extent, antimicrobials are also used to protect plants from diseases (mostly for rice, tomatoes, potato and citrus) [4, 5].

Several routes linked to animal husbandry, aquaculture and also plant production could lead to the spread of AMR in the environment. Antimicrobial residues and ARGs in plant foods could come from manure-amended cropland or sewage irrigation [6–10]. AMR propagation in the environment via field run-off could potentially affect both animal and human through consumption of drinking water and food [11].

**Fig. 1:**
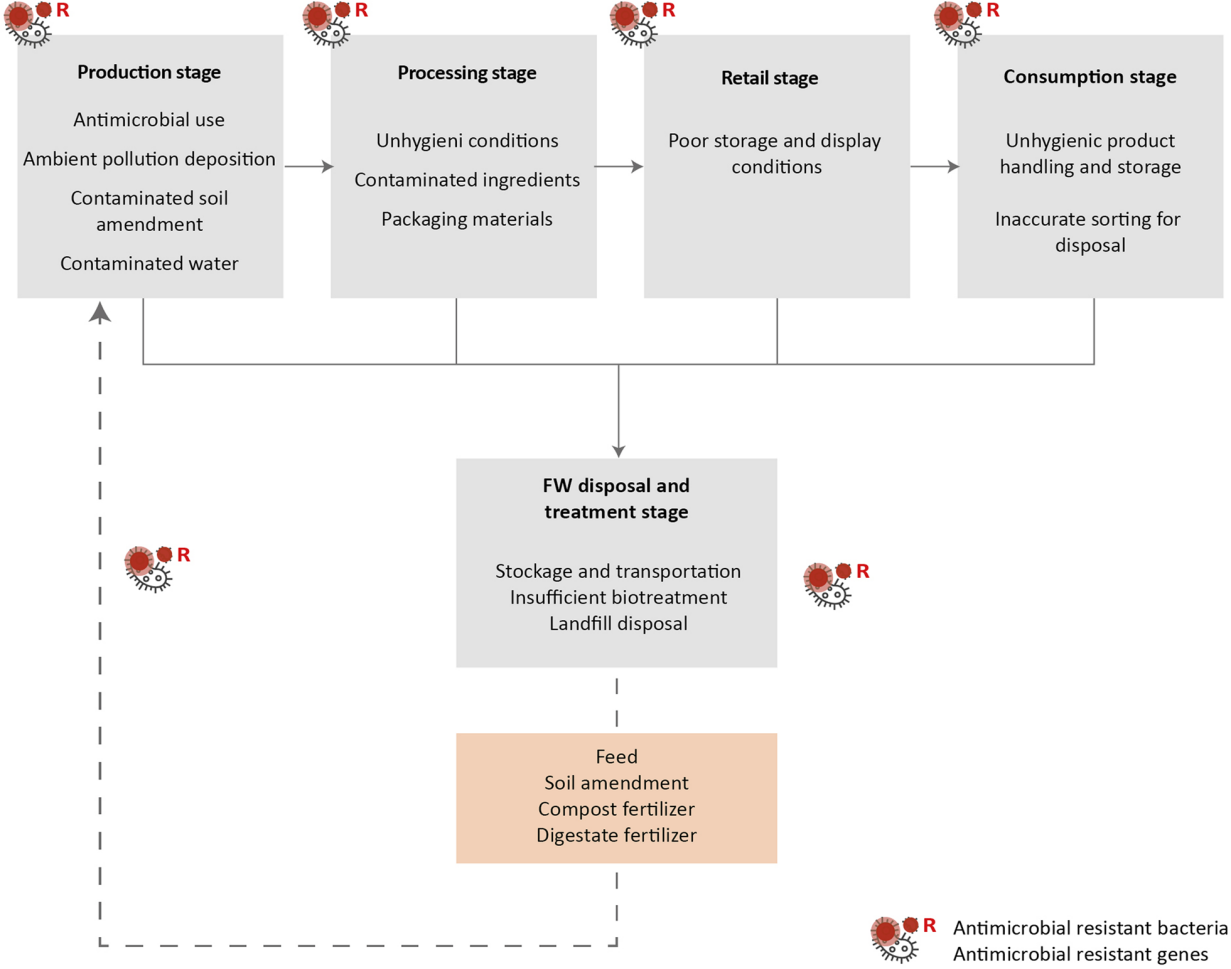
Potential antimicrobial resistance introduction/contaminations along the food value chain and through food waste materials. Adapted from [32]

AM introduced at the production stage may persist and drive the selection for ABR and ARGs throughout the food value chain. Then, potential contamination could also occur at various stages (Fig. 1). High resistance rate have been detected in several parts of the food supply chain [4]. Antibiotic residues could be found at retail and consumption stage, in various foods from animal origins (beef, pork and chicken meat, eggs, milk) but also in plant-based foods [12]. These levels are sometimes above the maximum residue limits, and residues of multiple antibiotics have been found in the same food products [13–15]. Food associated antimicrobial resistant bacteria (ARB) have been detected globally [16–24]. The prevalence of ARB is usually higher in meat [17, 20, 22]. However, it is noteworthy that some plant food such as carrots, lettuce, leaf and tomatoes also contain both antibiotic residues and ARGs [8, 25] and those plant foods can be consumed raw which can increase the risk of transmission to humans [26, 27]. Despite the presence of AMR in food products, the fate of AMR in food waste has been poorly characterized.

About one-third of all food produced for human consumption—1.3 billion tons—is lost or wasted, each year globally (FAO, 2019). Food Loss and Waste (FLW) has implications for food security, the environment and causing massive financial losses while squandering natural resources [28]. The goal established under the new Sustainable Development Goals global agenda is to halve food waste by 2030 [29]. In addition, the use of FLW in the circular economy is strongly encouraged for reuse (first for human then for animal), bioconversion, and energy recovery [29]. However, most food waste (FW) around the world is still disposed in landfills and improper use of FW in circular economy might lead to additional threats for animal and human health, including the spread of antimicrobial resistance through the environment [30].

This narrative review summarizes knowledge on AMR in food waste and its fate in various food waste management processes and explores the risk of AMR dissemination associated with FLW, that could compromise environmental, animal and human health. The scientific literature and data gathered show that FW may be an important but neglected source of antibiotic resistance that could contribute to environmental propagation of resistance if not properly disposed and treated. A better understanding of the connections between FLW and AMR would help to reduce the burden of AMR on the environment and optimize FLW management processes for a safer circular economy [31].

## Methods

This narrative review was conducted to explore the intersection between food loss and waste (FLW) and AMR, focusing on three core research questions: (1) Is AMR detected in food loss and waste? (2) What is the fate of AMR during different food waste management processes? (3) What are the potential environmental and public health risks associated with AMR spread via food waste pathways, including impacts on plants, animals, and humans? A literature search was carried out in two major databases, PubMed and Web of Science. No restrictions were placed on the date of publication, but only studies published in English were included. The search strategy combined terms related to food waste, antimicrobial resistance, and waste treatment or disposal processes, using Boolean operators (AND/OR). The following keyword groups were used in various combinations:

1. Food waste terms: “food loss”, “food waste”, “kitchen waste”, “canteen waste”
2. AMR terms: “antimicrobial resistance”, “AMR”, “antibiotic resistance”, “antimicrobial resistance genes”, “antibiotic resistance genes”, “ARG”
3. Waste treatment and disposal terms: “landfill”, “open dump”, “food waste disposal”, “food waste treatment”, “food waste biotreatment”, “composting”, “biogas”, “anaerobic digestion”, “aerobic fermentation”, “black soldier fly larvae composting”, “vermicomposting”, “fish silage”, “feed”, “rendering”.

In addition to the database search, the reference lists of all eligible studies were screened manually to identify additional relevant publications. Studies were included if they met the following criteria: peer-reviewed original research or review articles; examined AMR or ARGs in the context of food loss, food waste, or food waste-derived materials; investigated the presence, fate, or transformation of AMR/ARGs during waste treatment, disposal, or valorization processes; reported data, observations, or synthesized knowledge relevant to the environmental or public health implications of AMR spread from food waste. Studies focusing solely on clinical, veterinary, or general environment AMR unrelated to food waste streams, were excluded. The selection and synthesis of literature were guided by the review’s objective to map existing knowledge, highlight knowledge gaps, and propose future research directions concerning the fate and risks of AMR in the context of food waste management.

## Results

### AMR in the food waste

In this review, food waste refers to any kind of food (from animal and plant origin) and portions of food that are removed from the food supply chain to be disposed of or recovered. Some consist of edible parts but are unfit for human consumption (i.e.: leftovers from restaurants), some are inedible parts (i.e., carcass, slaughterhouse waste…).

Antibiotic residues as well as ARB and ARGs isolated from different types of food, can be found at all stages of the agrifood system: from primary production, to processing and retail, to consumption level and will inevitably be present in food wastes generated at those various stages [32] (Fig. 1). Food waste is a good substrate for bacterial growth and could foster the survival and growth of ARG-harboring bacteria. Subsequent contaminations of FW could also occur depending on FW management (collection, transportation, and pretreatment activities).

Zhao et al*.,* reported that the total antibiotic concentrations in the kitchen waste raw materials (25–207 μg/kg dry weight (dw)), before treatment, were in the same range as those reported in landfills (25–213 μg/kg dw), lagoon sludge from swine farm (3–127 μg/kg dw), and vegetable farmland soil (33–321 μg/kg dw) [33].

Studies revealed the prevalence of ABR, ARGs and mobile genetic elements (MGEs) in FW. In the US, a study showed that food waste collected from school, hospitals, grocery stores, restaurants or households contained ARGs [34]. The *tet(M)* and *blaTEM* were present in 96% and 97% of samples and with average abundance of (9 × 10^− 3^ copies/g dw) and (average of 6.8 × 10^8^ copies/g dw) respectively. In contrast, Eckstrom et al., reported the presence of ARGs in food waste from school, nursing home kitchens, grocery shops, but not from hospital kitchens [35]. ARG sequences related to aminoglycoside, tetracycline, and macrolide resistance were the most common. In another study, FW raw material (composed mostly from fruits and vegetables from grocery stores), showed absolute abundances ranging from about 2.96 × 10^6^ copies/kg for *ampC* to 3.58 × 10^9^ copies/kg for *sul1* before treatment [36]. Plant based substrates for anaerobic digestion (from sugar beet and corn silage) have also been found to contain ARBs and ARGs [37]. In India, a study on bacterial isolates collected from the dairy, hotel, meat, and canteen food waste samples, showed that the proportions of multidrug resistant isolates to a minimum three antibiotics were higher in canteen waste (42%), followed by dairy waste (36%) and hotel waste (33%) [38]. However, 11% of isolates from dairy waste and 8% from meat waste were resistant to about seven out of nine antibiotics. Strains from dairy waste (> 50%) were highly resistant to first-generation antibiotics; the strains from meat waste (> 50%) showed considerable resistance against second- and third-generation antibiotics. In another study, bacteria were isolated from different food wastes from meat slaughterhouses, dairy plants and restaurants, and were tested for antimicrobial resistances. More than 50% of all the strains were resistant to third generation antibiotics (vancomycin, neomycin and methicillin) [39].

Several studies showed that food waste arriving to composting or biogas plants contain significant level of ARGs before treatments, with important variations between studies. Different units are being used to report ARGs abundance. ARGs measured in FW from catering services were about 9.16 ± 0.32 log_10_ (copies/ml) (before anaerobic digestion) and 10.26 ± 0.16 log_10_ (copies/g) before composting [40]. The total ARGs abundance went from 10^4^ to 10^7^ copies/g DW [41], from 1.5 × 10^9^ to 2 × 10^10^ copies/g [42, 43], 1.4 × 10^8^ to 5.1 × 10^9^ copies/mL in food wastewater effluent before AD [44] and up to 1.6 × 10^13^ to 4.5 × 10^14^ gene copies/g dw in kitchen waste [33]. Zhao et al*.* reported that total ARGs abundance in FW materials before treatment were at comparable level or even higher of what is found in sewage sludge (approximately 5.1 × 10^11^ gene copies/g dw) or swine manure (approximately 2.5 × 10^11^ gene copies/g dw) [33]. However, in a study conducted Republic of Korea, ARGs relative abundance in food waste was one to two order of magnitude lower than in sludge or in manure with respectively 4.8 × 10^–4^, 4.6 × 10^–3^ and 2.5 × 10^–2^ gene copies/16SRNA [45]. Pan et al. reported much higher values in FW with ARGs relative abundance between 9 × 10^–2^ and 3.95 × 10^−1^ gene copies/16SRNA with multidrug resistance genes being the most abundant ARG type [46]. Wide variability of ARGs abundance in FW may occur as a result of type of raw material types (meat, crops), site-specific physical/chemical conditions that influence ARB growth and ARG propagation and attenuation dynamics. ARG abundances may also correlate with antibiotic use intensity and resulting residual antibiotic concentrations as it has been proposed for livestock waste [47].

**Fig. 2:**
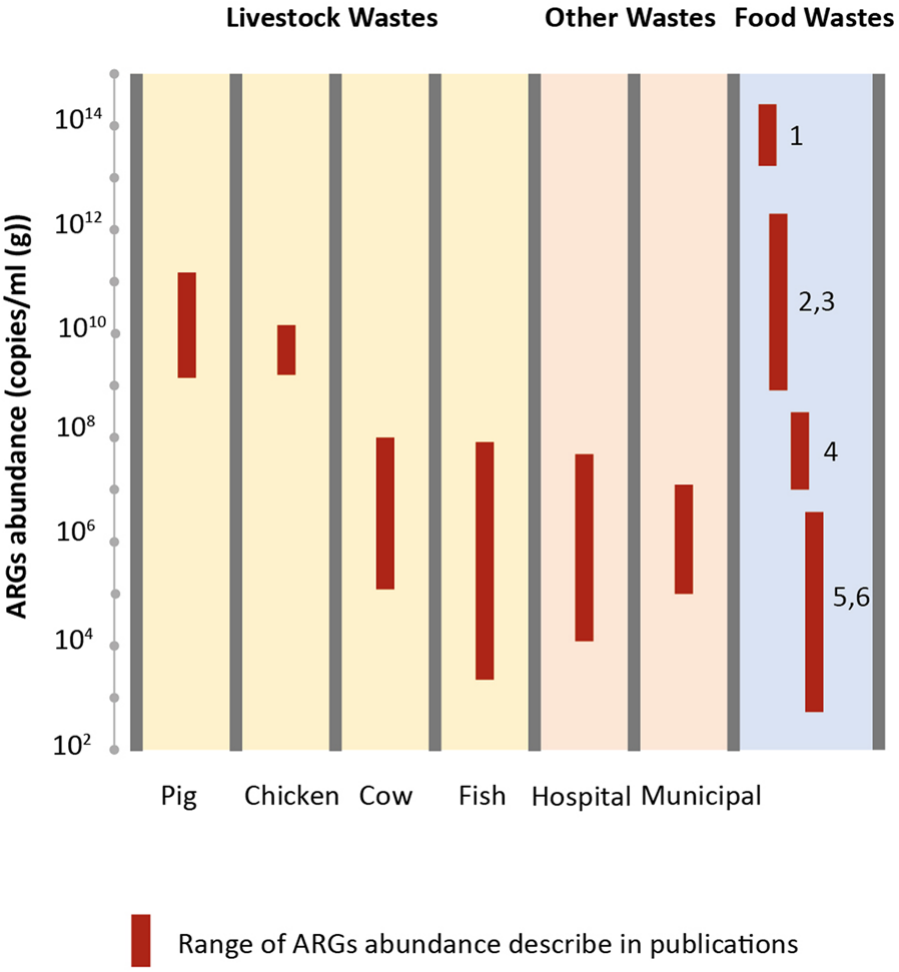
Comparison of ARGs abundance in various wastes (without treatment): livestock, hospital, municipal wastes and food wastes. Partially adapted from [47]. Data presented for food wastes are from 1 [33]: 2 [42]: 3 [43]: 4 [44]: 5 and 6 [36, 41]

In conclusion, despite important variability between studies, FW is a source of AM residues, ARB and ARGs, and the abundance is, in some cases, comparable to other type organic wastes, as livestock wastes and sludges, that have been for a long time identified as drivers for AMR dissemination in the environment [45, 47, 48] (Fig. 2). Specific attention to AMR risk in the context of FW disposal and treatment is needed.

### AMR fate in food waste management processes: generalities

**Fig. 3:**
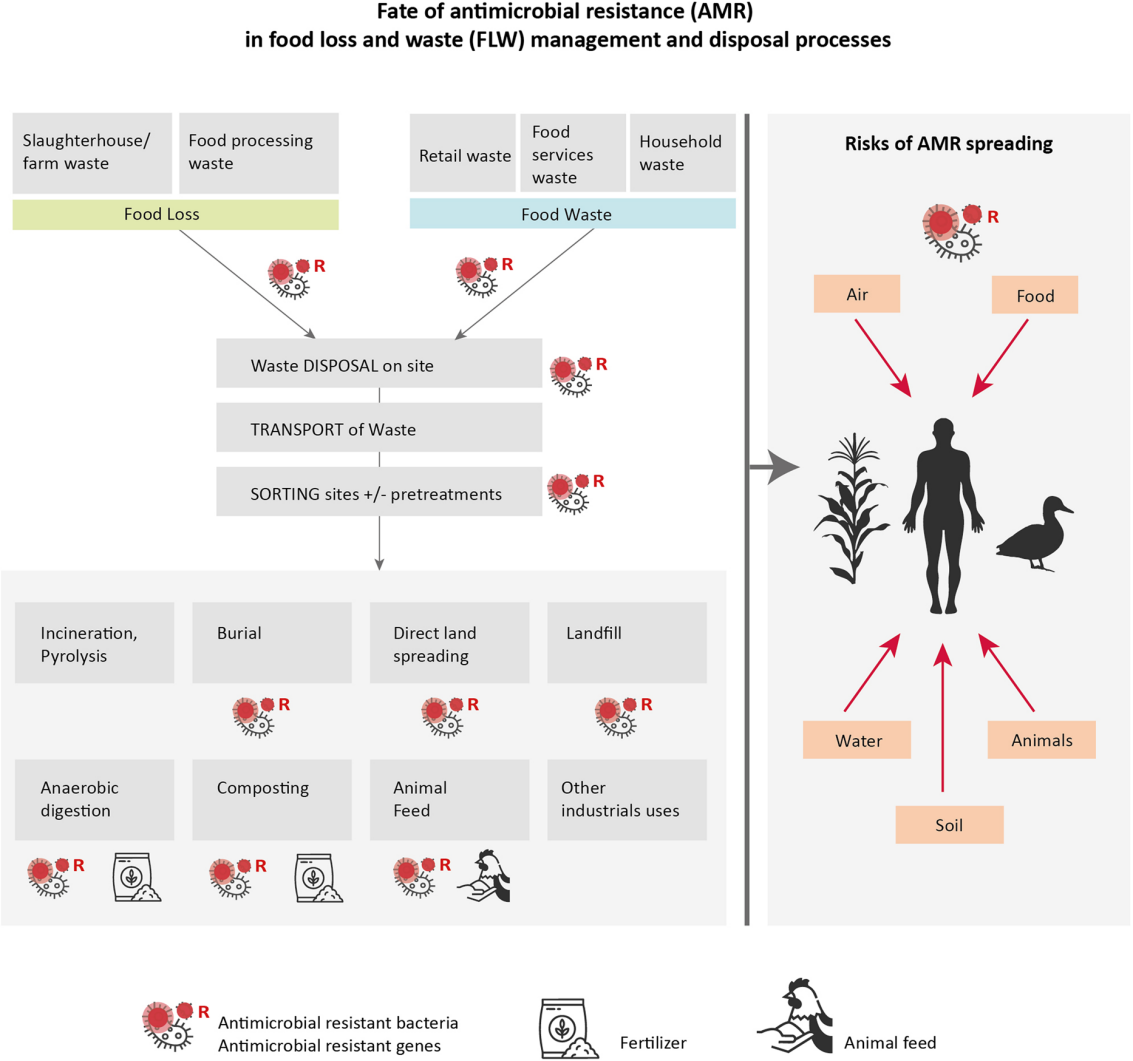
Overview of food management processes and potential routes of AMR spreading. The top part corresponds to wastes generated at different steps of the food value chain. Green corresponds to food loss and blue to food waste. In grey, the different stages for food loss and waste management. FW could be treated on-site, or transported to sorting sites depending of the origin of FW (industrial, retail or household) and infrastructures. FW is then disposed or treated. This mapping is not exhaustive. The left part shows the potential dissemination routes of AMR and pathogens

To evaluate the possible contribution of FW to AMR risks for the environment, animals and in humans, it is essential to understand how food loss and food waste are disposed of and treated and how these processes influence antibiotic residues, contamination and growth of pathogenic microbes and the spread of ARGs (Fig. 3). There is a food waste management hierarchy: prevention of FLW, preparation for re-use, recycling, other recovery (i.e. energy recovery) and landfill treatment [49]. If food waste is not fit for human or animal consumption, it can be diverted for other uses, such as nutrient, material and/or energy recovery. The quality and nature of food waste (stage of collection and safety) as well as infrastructures in place, determine the type of treatments that could be applied (Tables 1).

**Table 1:** Types of food management processes and impact on antimicrobial resistance

Category	Methods	Purpose/Output products	Impacts related to antimicrobial resistance
Valorization pathways (energy, material, and nutrient recovery)	Transformation into value-added products (for feed and food)	Nutritional and economic valorization	Variable depending on hygienization and processing method
	Aerobic fermentation /Composting	Production of organic fertilizer	Partial reduction of ARGs/ABR, dependent on operating conditions
	Vermicomposting and black soldier fly composting	Production of fertilizer and larval biomass	ARG/ABR reduction dependent on operating conditions
	Anaerobic digestion and anaerobic co-digestion	Production of renewable energy (biogas, hydrogen) + digestate used as organic fertilizer or animal bedding	ARG/ABR reduction dependent on operating conditions
Disposal methods	Incineration	Waste destruction + possible energy recovery	No risk—due to very high temperature
	Combustion	Destruction with energy recovery	No risk—due to very high temperature
		Production of biochar for soil amendment	No risk—due to very high temperature
	Burial	Disposal (mainly for animal carcasses)	May lead to environmental contamination
	Landfill	controlled disposal	Partially controlled ARG/ABR dissemination
	Open dumping	Uncontrolled disposal	High risk of ARG/ABR dissemination

ARB: Antimicrobial resistant bacteria; ARGs: Antimicrobial resistant genes

Disposal of FW in dumps and landfills represents the first disposal method. Many countries are adopting ambitious programs to encourage and promote nutrient and energy recovery to reduce or eliminate food waste from landfill (i.e.: Republic of Korea, EU countries, Australia). Within the circular biobased economy, FLW represent a huge feedstock for improved food security and health.

Food wastes are nutrient rich environments and could have high water content. These complex matrices spoil easily and are favorable to microbial growth. Micro-organisms, including bacteria and fungi, present in the raw material or coming from contamination. Fate of the ARGs in food waste and FW processing is influenced by several factors: (1) **abiotic conditions:** (i.e.: temperature, oxygen, level of organic matter, pH, presence of additional contaminants). Multiple non antibiotic molecules could influence susceptibility of bacteria to antibiotics [50]. Among them disinfectants, heavy metals (HMs) and microplastics (MPs) are those most studied and those that are susceptible to be found in FW [51–55]; **(2) microbial populations dynamics and (3) horizontal gene transfer (HGT) estimated by mobile genetic elements (MGEs):** (i.e., transposon, integron, conjugative plasmids…).

Similar biotreatments could be used to manage various types of waste. However, not all results related to other types of organic waste can be extrapolate to FW. Few studies showed that ARGs removal was less efficient in FW than for other organic waste [56, 57]. Ma et *al*. compared ARGs dynamics and the mechanisms driving them during the composting of different types of organic solid wastes: pig manure (PM), kitchen waste (KC), and sewage sludge (SG) [57]. Among the three organic wastes, the pig manure had the highest ARGs abundance (5.49 × 10^−1^ copies per 16S rRNA), but the ARGs were largely removed (71%) during composting. On the contrary, ARGs abundance was increased 5.9-fold in KC and 1.3-fold in SG. Sulfonamide in KC (38-fold) and tetracycline in SG (fivefold) showed the greatest increases. The authors concluded that not all substrates can be considered equivalent for AMR removal treatments and more attention should be paid to the ARGs risk of kitchen waste composting [57].

### Transport and storage before food waste treatments

Transport and storage before waste treatment is a critical step as microbial population present in FW might increase significantly as well as the number of ARGs and horizontal gene transfer. This period could also be critical for cross-contamination.

Lin et al*.* have tested, in laboratory conditions, the impact of short-term storage (from 1 to 25 days, at 25–28 °C) on the fate of AMR in four food wastes (cooked vegetable, meat, fish, and rice) [43]. During this short-term storage, the absolute abundances of ARGs significantly increased in the four different wastes. The number of ARGs and MGEs first increased, then stabilized and finally declined in all food waste samples. But types of ARGs and kinetics were different depending on the type of food. Fish waste samples harbored the highest diversity and abundance of ARGs, followed by vegetable, meat, and rice waste samples. Interestingly, ARGs abundance was of the same order of magnitude to those previously reported in food waste processing plant before composting (10^9^ to 10^10^ copies/g) [42]*.* Pathogens in animal food waste (i.e., meat and fish) present a higher ARGs diversity, compared to vegetable and rice. Overall, animal-derived food wastes, especially fish samples were more harmful considering the high load of ARG and pathogens they accumulated during spoilage. The authors recommended: rapid food waste collection, transportation, and effective pre-treatments to reduce microbial and ARGs load of the feedstock for subsequent food waste management processes.

The storage period is also critical, because if the food waste is poorly stored it could lead to bacterial dispersion (via water or airborne particles) and eventually be eaten by animals or even by humans, with direct health risks. More data are needed.

### Aerobic fermentation (composting) of food waste

Composting is considered as an environmentally friendly, and cost-effective bioremediation technology. Food waste should be uncontaminated with other materials (plastics, metals) and toxicants. Composting of FW produces an organic fertilizer that can be used as a nutrient-rich soil amendment in agriculture. However, there are challenges around the safety of food waste compost. The risk of AMR spreading through composting will greatly depends on various parameters in the aerobic fermentation process.

#### Factors influencing ARGs fate in food waste composting

Composting is a complex biotransformation process that is deeply influenced by both biotic (mainly bacterial population, MGEs) and abiotic factors (temperature, pH, Carbon/nitrogen (C/N)).

Liao et al*.* studied AMR fate in a full-scale aerobic composting plant in China [42]. In this site, raw composting materials consisted of pre-treated FW (deoiled) and tobacco powder. The absolute abundances of ARGs and MGEs increased during composting, but their relative abundances decreased, and fewer bacteria carried ARGs and MGEs at the end of composting [42]. High composting temperatures reduced the number of initial ARG-associated bacterial taxa, but data suggested that ARGs were likely maintained in other bacterial taxa that resisted periodically high temperatures, potentially through HGT as indicated by strong positive correlations between ARGs and MGEs. Both physicochemical composting properties and MGE abundances had equally strong direct positive effects on ARG abundances. Overall, this study showed that traditional composting reduced ARGs and MGEs in FW but not efficiently enough to remove the risk of AMR spreading.

In a study of on-farm traditional composting of FW, there was no reduction in the number of ARGs genes in the final compost [35]. Other studies showed an increase in ARGs after the composting process of FW (from catering services): 5.9-fold (kitchen waste [57]), 8.2-fold (co-composting swine manure and FW [58]), 9 up to 66-fold [40]. Optimization of composting strategies are needed to minimize ARG release from composted FW into the environment.

#### Temperatures, material movement and residence time

Parameters as temperature, material movement, residence time and ventilation, can be optimized to improve the quality of the final product and reduce the time of composting. Different composting processes have different impacts on ARGs.

In a study, Zhao et al*.* collected kitchen waste samples [33]. Four different types of composting were tested: aerated static pile composting (ASPC), dynamic pile composting (DPC), solar-assisted composting (SAC) and mechanical composting (MC). Composting processes differed in their material movement, form of the material stacking: pile or vessels, ventilation. This influences residence time and temperature of the compost (Table 2).

**Table 2:** Four different types of composting tested on kitchen waste

Processes/characteristics	Material movement	Material stacking	Ventilation	Residence time (days)	Temperature
ASPC	Static	Pile	Positive pressure	50	** + + **
DPC	Dynamic	Pile	Natural	30	** + **
MC	Dynamic	In vessel	Positive pressure	15	** + **
SAC	Static	In vessel	Positive pressure	45	** + + + **

*ASPC* Aerated static pile composting; *DPC* Dynamic pile composting; *SAC* Solar-assisted composting; *MC* mechanical composting. [33]

Despite composting, antibiotic residues and ARGs persist respectively at 22.9–122.3 μg/kg (dry weight) and 10^12^–10^14^ copies/kg (dry weight). But there are major differences depending on the composting processes. Static composting (ASPC, SAC) gave better results at removing antibiotics and some ARGs compared to dynamic composting. There was also strong variability depending on the class of antibiotics. After composting, the total ARG abundances declined significantly in all the four processes, mostly because on the most abundant ARGS, the tetracycline resistant genes that showed a reduction of more than 99.9%. Among the four composting processes, only SAC could degrade all types of ARGs with a reduction of more than 98% of tetracycline, quinolones, and macrolides resistance genes and 43% of sulfonamides resistances genes. Overall, pile composting (ASPC and DPC) induced a significant enrichment of ARGs (> 360% increasing rate except for tetracycline ARGs). For example, in DPC, quinolones macrolides, sulfonamides ARGs were enriched by 837%, 4425%, and 8534%, respectively.

Maintaining a high temperature (higher in static composting), a low pH in the up-temperature phase, and suitable rate of water loss can serve as a good composting strategy to reduce antibiotics and ARGs in kitchen waste. In conclusion, it is essential to optimize composting processes (e.g., static, higher temperature, and in vessels) to reduce AMR risk.

Yang et al*.* showed, on composting of swine manure: food waste: cornstalk mixture, that increasing high temperature duration (from 5 to 9 days in two stages: day 3–6, 55 °C and day 7–10, 70 °C)) improved elimination rates of ARGs and MGEs, achieving a reduction of 85% and 97% respectively [58]. In contrast, in the same study, ARGs increased by 8.2 times by the end of the process with conventional composting.

Hyper thermophilic composting technology can raise the maximum temperature of composting to about 90 °C without external heating, which is 20 °C–30 °C higher than traditional composting. On FW, hyper thermophilic composting has showed a good level of removal for most tested ARGs, except for *ampC* [36].

Batch-fed composting (BFC) is different from dynamic composting and aerobic composting in that the process has only infeed and no discharge until the end of composting. It has a long residence time which allows the material to be fully decomposed [59]. FW generated from the canteen of an industrial park, was mixed with sawdust and bacteria were added in the started phase. The analysis ran for 21 days. In this process, temperature increased rapidly, exceeding 60 °C within 3–5 days. Results showed that the continuous high temperature provided by BFC was beneficial in reducing ARGs [59]*.*

#### Pre-treatments

Various physical pre-treatments, microbial inoculants and additives could be used to both shorten composting period. They could influence ARGs abundance.

Li et al*.* have assessed the impact of two pre-treatments, **biodrying and mature compost inoculation** on AMR fate [60]. Biodrying by reducing moisture content of FW, could accelerate composting maturation. Mature compost is used to accelerate the composting process. By using those two pretreatments, the final maturity of compost was achieved after 9 days of composting (compare to 15 up to 50 days in other processes). In addition, both pretreatments could modify ARGs dynamics as it increased temperatures. Those conditions inhibit the proliferation and spread of potential bacterial hosts of ARGs and integrons. Data showed a significant reduction of the absolute (from 72 to 99%) and relative abundances (0.3–2.4 logs) of nearly all tested ARGs especially *ermB*, *tetM*, *bla*_*CTX-M*_ and *bla*_*OXA*_. The authors concluded that biodrying and addition of mature compost could mitigate ARGs dissemination and achieve compost maturity in a short time, however no direct comparison have been made with control setting to evaluate the impact of the addition of mature compost for the ARGs load.

Ahmed et al*.* tested the effect of initial enhancement in composting temperature (high temperature HT (60 °C) in the 5 first days) in a co-composting of swine manure, FW from canteen and corn stalk with a conventional composting (NT-without initial heating) [61]. ARGs were reduced by 49.3% and 79.4% with NT and HT respectively. In addition, there was no rebounding of any ARGs after 30 days with HT. MGEs were reduced by 68.1% and 93.6% in NT and HT. ARGs variation was directly related to composting temperature. Bacterial communities were mainly responsible for the enrichment of ARGs in NT, whereas it was directly affected by MGEs in HT. Overall initial composting step at 60 °C significantly reduced ARGs associated risk.

**Enzymatic pre-treatments** also help structural breakdown of food constituents and accelerate composting. Du et al. shown that an in situ multienzyme treatment can also reduce up to 90% of ARGs [62].

**Addition of microbial agents** are commonly used for the large-scale composts, but one study showed that it might enhance the persistence of antibiotic resistance genes in aerobic compost of food waste. Zhang et al*.* tested two kinds of microbial inoculants on food waste composed of vegetables, staple food, fruit peel, meat, eggshells and bone and mixed with sawdust in compost [63]. The first inoculant VT-1000 was composed of lactic acid bacteria, *Bacillus*, yeast, mold, *Actinomyces* (≥ 2 × 10^8^ CFU/mL) and VT-1020 included side spore bacillus, yeast, lactic acid bacteria, (≥ 0.50 × 10^8^ CFU/mL). Result suggested that the addition of microbial agents was detrimental to the reduction of ARGs type in compost solid. A portion of tetracycline resistance genes (i.e., *tetD* and *tetV*) and multidrug resistance genes (i.e., *MuxB* and *MuxC*) cannot be removed by the treatments involving the addition of microbial agents causing ARGs to develop tolerance to high temperatures. One of the explanations seems to be that the addition of microbial inoculant reshaped the compost community structure with the consequence of increasing the effect of the bacterial community on ARGs.

#### Co-composting

Co-composting is very communally used to optimize parameters such as moisture and C/N. Bulking agents such as rice husk or sawdust are excellent regulators of free air space and moisture content, with greater stability and capacity to reduce leaching than other materials [59]. However, the type of bulking agents needs to be optimized for each type of waste.

In addition, FW could also be composted with other organic wastes. One study followed ARGs in co-composting of FW (coffee) with chicken manure and the bulking agent urban green waste. Interestingly, in this study urban green waste, and not manure, was the main source of ARGs [64]. Adding up to 40% of cattle manure to FW was also shown to improve composting and AGRs removal [65].

Chen et al*.* tested in lab conditions the effects of co-composting of FW with sewage sludge on ARGs [66]. In this experimental set-up, antibiotics were added at the initial stage. Data showed that co-composting effectively removed fluoroquinolones and macrolides but was poorly efficient in removing sulfonamides. The co-composting affected ARGs differently. After 28 days, there was a decreased abundance of *sul3, sulA, qnrB, qnrS,* and *ermB* (0.93–2.77 logs), while the abundance of *sul1, sul2, qnrD, ermC,* and *ermF* increased. Total organic matter was found to be the most important factor for the variation in the ARGs abundance.

Co-composting of food waste digestate produced from anaerobic digestion with Chinese herbal medicine residues showed [67]. Great removal of remaining antibiotics by 83% and effective acceleration of the maturity of FWD composting. However, it induced a significant enrichment of ARGs and MGEs including *frA1, tetX, blaTEM, InuB-01*, *aadA2-02* and *IntI-1*.

### Anaerobic digestion of food waste

In the anaerobic digestion (AD) process, organic matter is degraded anaerobically by a variety of microorganisms working in synchrony. The final products from this degradation are biogas (for production of electricity, heat and vehicle fuel) and digestate that is commonly used as fertilizer, animal bedding or even as building material. The composition of the raw material used, combined with chosen operational parameters, influences both biogas yield and the composition and quality of the AD digestate. However, AD is rarely optimized to remove AMR [40, 46]. Parameters such as pre-treatments, temperature, residence time, solid content, and the number of stages, could also drastically influence the ARG abundances in digestate [68].

#### Pre-treatments before anaerobic digestion

There are various pre-treatments: physical, chemical and biological. In their review on sludge, Han et al*.* showed that there is a trade-off between yield and ARGs removal. In most cases, the pretreatment method and the operational conditions for maximum methane production may not achieve optimal ARGs removal [69].

FW contains complex components such as starch, cellulose and protein that are difficult to be directly used by methanogenic microorganisms. Without pretreatment, it could lower AD efficiency (generally 30–50%) of food waste. Enzymatic methods promote hydrolysis of food waste, and in turn to enhance the efficiency of downstream AD [70, 71]. Both commercial enzyme cellulase (Viscozyme L) and protease (Flavourzyme) were used to hydrolyze and solubilize stable polymer networks of proteins and celluloses in FW. Enzymatic pretreatments reduced microbial diversity and various potential ABR and decreased ARGs transmission including *tetW*, *tetQ*, *vatB*, *CfxA2*, *bacA*, *ermF* and *mefA* by modifying genetic expressions. Overall, enzymatic pretreatments were found to decrease AMR risk from anaerobic fermentation from 13 to 24.5% [71].

Less costly than commercial enzyme, the use of fungal mash containing multiple hydrolytic enzymes and produced in situ from FW was also shown to accelerate food waste disintegration and AD efficiency [70]. In addition, most of ARGs (*sul1*, *ermB*, *mefA*, *tetA*, *tetQ*, *tetW*, *ampC* and *qnrA)* were reduced by 45–94% after 24 h enzymatic pretreatment except *sul2* and *bla*_*TEM*_, which increased by 11% and 19%, respectively. The total tested ARGs and *intI1* were respectively further removed by 44–55% and 21–73% in subsequent AD process. The evolution of microbial community composition and horizontal gene transfer (here *intl1*) were the common driving forces for the variation of most ARGs.

Microwave pre-treatment has also been used on sewage sludge and food waste [56]**.** Microwave-pre-treatment of sewage sludge provided better ARG removal than pre-treatment of food waste. ARG removal was higher in microwave-enhanced co-digestion than mono-digestion of sewage sludge.

#### Factors influencing fate of ARGs in AD of food waste

Various operating parameters can influence the fate of ARGs in AD. Temperature has been the most widely studied process parameter. Overall, numerous studies showed that higher temperatures (55 °C) have been shown to have a better impact than mesophilic processes (between 20 and 45 °C) to reduce ARGs in AD [68]. Data showed that feedstocks for AD will greatly influence the optimum conditions for ARG removal efficacy [72]. The removal efficiency can also differ from one targeted ARG to another. Two studies, on FW from canteens, showed a decreased of ARGs with thermophilic conditions [73, 74]. The ratio of ARGs removal in thermophilic condition (55 °C) was 86.6% (± 0.7%) compared to 31% (± 0.4) in mesophilic digestion (35 °C) [74].

#### Co-digestion

Mono-digestion of FW could fail due to the accumulation of organic acids and limitation of some nutrients [75]. Anaerobic co-digestion (AcoD) of FW is usually conducted with other substrates, as sludge, manure or straw, to enhance the efficiency of AD, to adjust C/N, pH, moisture content and to enhance AD efficiency [76]. Sources of feedstock and mixing ratio have different impacts on ARGs removal. Some materials could contain antimicrobials or high ARGs loads (sludges, manures), or on the contrary others might have specific properties that could reduce ARGs (medicinal herbs, paper). In a study, co-digestion of FW with paper and cardboard in a lab-scale solid-state, ARGs relative abundance and diversity were significatively decreased after 6 weeks of digestion [41].

On the contrary, sludge used for co-digestion and co-composting with FW could be a source of significant amounts of antibiotic residues (sulfonamides, quinolones, macrolides, tetracyclines), as up as 4.3–21.3 mg/kg [77, 78]. Those levels of antibiotic concentrations could have an impact on both AD efficacy by disturbing the bacterial populations in the digester and ARGs abundance and expression. Wang et al*.* tested the effect of 5 and 50 mg/L of tetracycline, sulfamethoxazole and erythromycin on AcoD of FW and sludge. During 70 days of AcoD process, antibiotics were found to inhibit and delay both maximum daily methane and cumulative methane yields except for 5 mg/L erythromycin. Antibiotics induced the declined abundance of acetogenic *Proteiniphilum* and hydrogenotrophic *Methanobacterium* in antibiotics-added digesters, resulting in the inhibition of FW hydrolysis process and delayed the methanogenesis [79]. In the same conditions, the authors also found that the antibiotics increased ARGs abundance by influencing microbial population and enriching integrons. In another study, with AcoD of FW (from canteen) with chicken manure or FW with water activated sludge (1:1), ARGs removal was more effective in mono-digestion of food waste than its co-digestion with chicken manure and waste activated sludge [80]. Manure and sludge pre-treatment to reduce antibiotics/ARGs concentration might be needed.

#### Two-stage (acidogenic-methanogenic) anaerobic digestion

The process of AD involves interaction between different types of micro-organisms, fermentative bacteria (acidogenic) and the methanogens, that have different nutritional requirements and growth kinetics. The two stages of the AD process, an acidogenic phase followed by a methanogenic phase have been developed to provide optimal growth conditions for each microbial group. Jang et al*.* showed that two-stage AD was more efficient than one-stage AD for the removal of ARGs and *intl1* present in food waste wastewater even if some ARGs persist in the effluent even in two-stage AD [44].

#### Additives

Conductive materials (e.g., activated carbon, biochar, magnetite, etc.) in AD improves methane production and also influence ARGs removal [76].

Zhang et al. showed that activated carbon in AD on FW reduced ARGs, including *tetA, tetM*, *tetW, tetO, tetQ, sul2* and *tetX*. Activated carbon has high porosity and a large surface area. It decreased bacterial pathogens by 18%. Bacteria adsorbed on activated carbon might have a reduced bacterial motility which could decrease HGT. However, it did not improve the removal of other ARGs (*sul1, sul2, cmlA*, and *floR*), and *intI*. [81]. Powdered activated carbon (15 g/L) decreased ARGs in mono-digestion of FW but not in co-digestion with chicken manure or wastewater sludges as both contain significant amount of antibiotics [80].

In another study, in the co-digestion of food waste (university canteen in Singapore) and chicken manure, powdered activated carbon removed different classes of antibiotics (e.g., lincomycin, ciprofloxacin, erythromycin, etc.) probably by providing efficient adsorption of various chemical, which promote ARGs removal [82].

Addition of nanoscale zero valent iron (nZVI) (5 g/L and more) in AD thermophilic co-digestion of sludge and kitchen waste (canteen university) reduced the amount of *tet* ARGs. Interestingly, *tet* genes encoding different resistance mechanisms behaved distinctly in AD [83].

In another study, the effect of nZVI was assessed in co-digestion of FW (canteen, university) with sewage sludge at thermophilic (55 °C) or mesophilic (35 °C) conditions. The highest ARGs removal ratio (> 85%), especially on tetracycline ARGs (*tetO, tetQ, tetX*), was obtained in the presence of 2 g/L of nZVI and in thermophilic conditions (compared to mesophilic conditions). Redundancy analysis indicated that microbial community was the main factor that influenced the fate of ARGs [74]. Systematic optimization of all parameters is needed to ensure efficient ARGs removal.

### Comparing food waste biotreatments for AMR removal

Few studies have made direct comparison of the biotreatments of FW on AMR removal. Costa et al*.* compared the impact of three types of treatment on AMR fate in FW: (1) Thermal treatments: both pyrolysis and incineration, (2) Hyperthermophilic composting for 12 days under exogenous heat at 90 °C in an oven, (3) anaerobic membrane bioreactor [36]. The solid food waste was primarily consisting of fruits and vegetables from local grocery stores. Incineration and pyrolysis were the most effective techniques for ARG removal. All measured genes were below detected levels. Hyperthermophilic composting removed six out of the eight ARGs tested. *ermB, ermF, oxa-1, tetW, tetO*, and *sul2* genes to below detection limits but only partially removed *ampC* detected at 2.4 × 10^6^ copies/kg and *sul1* at 1.0 × 10^8^ copies/kg after treatment. With the anaerobic membrane bioreactor treatment, high log removal of ARGs was achieved in effluents. Genes *ampC, oxa-1, tetO*, and *intI1* were below detection limit for all treatment conditions. Few ARGs persisted in the effluent (*ermF, sul1*, and *sul2*), *sul1* remained the highest among treatment conditions and *ermB* was the gene that accumulated the most under the same conditions. Persistent ARGs likely result from the selection of ARG bacterial hosts able to resist high temperatures such as members of thermophilic phyla (e.g., *Thermi* and *Firmicutes*) and heat tolerant MGEs. However, with anaerobic membrane bioreactor treatment, biosolids retained all targeted ARGs in all operating conditions. Biosolids achieved partial removal of *oxa-1* and *intI1* gene but accumulated *ampC, sul2, ermB, tetO*, and *sul1*. *EmrB* was detected at 1.0 × 10^10^ copies/kg biosolids. Authors suggested that some treatments could be combined. The anaerobic membrane bioreactor when used to recover energy from food waste, could produce a high-quality effluent, and produced biosolids that might have to be pyrolyzed to remove remaining ARGs while producing biochar.

Wang et al*.* also compared the initial and biologically treated FW in two major FW treatment systems with aerobic fermentation (AF) and anaerobic co-digestion (AcoD) processes [40] in China (catering waste but from different sites). In AcoD treatment system, anaerobic activated sludge was added to FW. In AF, the treatment process was operated in 60–80 °C for 12–14 h with addition of microbial agents to promote biotransformation processes. The relative abundances of integrons and targeted ARGs significantly increased in the two biotreatment systems for all samples [40]. The total ARGs relative abundance respectively increased 66.88 ± 87.34 and 27.23 ± 24.30 times in AcoD and AF systems. The relative abundances of *mexF, sul1, aadA, strB, ermB, blaOXA, intl1* and *intl2* showed increasing trends from initial FW to treated samples in both biotreatment systems. However, the abundances of *mefA* and *blaTEM* from initial FW to treated samples were only increased in AF system, while decreased in AcoD system. The higher enriched *sulR* and *tetR* in AcoD system came probably from the sludge inoculum applied in initial stage that could contain high levels of antibiotics, heavy metals and increase selective pressure for ARGs. Compared with AcoD system, no additional contaminant was transported into AF system, and the potential risk might be relatively lower. Potential bacterial host also influenced ARGs during FW treatments: Firmicutes were highly abundant in initial FW, Proteobacteria in AF-treated samples and Bacteroidetes in AcoD-treated samples. Data showed that integrons (*intl1* and *intl2*) had a pivotal role on the occurrence and dissemination of ARGs in FW treatment systems. The authors evaluated the total discharge of ARGs at 10^7^ copies per day and up to 10^8^ copies/day respectively for AF and AcoD [40]. Based on those data, AF gave better results than AcoD.

Li et al*.* have studied the fate of AMR in two full-scale FW leachate treatment processes [84]: (L) an anoxic-aerobic-anoxic-aerobic system and (W) an anaerobic-anoxic-aerobic system. Before treatments, the absolute abundance of total ARGs in raw leachates ranged from 2.3 × 10^7^ to 2.1 × 10^8^ copies/mL in L system and from 1 × 10^7^ to 2.8 × 10^9^ copies/mL in W system. Four genes, including *mexF*, *blaCTX*-M, *sul1* and *intI1* were found with higher abundance across all samples. Following the treatments, the total abundance of ARGs decreased by 76% (from 1.1 × 10^8^ to 2.5 × 10^7^ copies/mL) and 98% (from 5.6 × 10^8^ to 1.04 × 10^7^ copies/mL) in L and W system respectively. Nearly all absolute abundances of individual target ARGs were effectively reduced in the effluent of L (32.8–99.8%) and W system (69.0–99.9%), respectively. The absolute levels of *intI1* and *intI2* declined above 99% in effluent of W system, compared with raw leachate. In contrast, *intI1* was enriched by 3.9 times in the effluent of L system. ARGs were enriched in sludge samples: to 7.9 × 10^10^ and 2.2 × 10^10^ copies per gram in L and W. However, the relative abundances of ARGs and integrons were notably increased, meaning that a higher proportion of bacteria acquired ARGs and integrons during biotreatment processes. There were different profiles of ARGs between the two processes. In the L system, ARGs were positively influenced by physico-chemicals parameters and integrons but mostly by integrons in the case of W. Overall, estimation of the discharge of effluent and sludge included high levels of ARGs (5.1 × 10^14^–4.8 × 10^15^copies/d), integrons (1.1 × 10^14^–6.0 × 10^14^copies/d) and potential pathogens (such as *Pseudomonas* and *Streptococcus*). Despite high discharge, the W (with anaerobic digester) process might have better remove effect than the L process on the occurrence of ARGs and integrons.

Yang et al*.* studied the ARGs risk associated with FW compost and FW digestate used as fertilizers to grow pakchoi in laboratory conditions. Application of FW compost resulted in a higher diversity of ARGs in soil and endophytes [85]. In those conditions, FW compost could represent a higher risk, but results should be taken with caution as the raw FW used for composting and AD were different.

### Risks of AMR spreading via food waste biotreatments

There are several ways in which improper treatments of organic waste could contribute to AMR spreading in the environment and ultimately to animal and humans contributing to the rise in resistant clinical infections (Fig. 3).

1. When the final products of biotreatments are used as fertilizers. They could introduce AMR into the environment (soil, water, wildlife) and in the food chain through plants and crops. It could return to humans through water and food, and eventually through direct contact with soil and wildlife.
2. During transportation and the waste treatment processes, AMR can contaminate nearby environments, animals and humans through aerosols exposure, leakage and effluents.

However, very few studies have measured AMR spreading from FW biotreatment by-products.

#### AMR in biofertilizers from food waste compost and digestate

Data show that the final product resulting from composting, when used as fertilizer, could contain ARGs [40, 85–87]. In Ireland, a study showed that the antimicrobial resistance profile of a city food waste compost was significant, and that bacterial populations present in the compost had genes encoding resistance to several classes of antibiotics, which are important and sometimes critical to human health. None of the compost samples tested displayed sensitivity to all tested antibiotics (lincomycin, tobramycin, minocycline/amoxycillin, ciprofloxacin, florfenicol) [86]. In China, a study showed that all samples of commercial organic fertilizers contained high abundances of ARGs encoding resistance to tetracycline, sulfonamide, aminoglycoside and macrolide. The raw material had a significant effect on the ARG abundance, with the presence of animal feces generally having higher loads of ARGs than those from non-animal raw materials such as food waste [88]. This was consistent with a study in the US, where backyard composts, composed mainly of food and garden wastes, had lower relative abundances of ARGs than commercial composts (made from animal manure) [87].

Studies showed that FW digestates from AD**,** that can be used as fertilizers, also contained ARB and ARGs [40, 85, 89–91]. Studying products from AD using different substrates from biogas plants in Poland, Wolak et al*.* showed that digestates contained also high levels of antimicrobial residues, particularly doxycycline and tetracycline at concentration ranging from 218 ng/g to even 1282 ng/g and 464 ng/g to 1164 ng/g respectively. ARGs were detected in all digestate samples at concentrations of 1.16 × 10^3^ to 2.25 × 10^6^ copies/g of digestate [90]. Sun et al*.* compared biogas digestates from two processes, using animal manure (AM) or FW as substrate. Among isolated species, many were multi-resistant. Both manure digestate and FW digestate contained bacterial species resistant to all antibiotics tested (11 out 12), except gentamicin. Interestingly, a higher level of resistance was displayed by the FW isolates. One explanation could be that there is a higher antibiotic pressure in FW compared with AM digestate [89].

Used as fertilizer, food waste compost and digestate could introduce ARGs into the agroecosystems. Pan et al. compared ARGs in FW, digestate and soil with or without digestate. Overall, the total relative abundance of ARGs in soil supplemented with digestate was lower than that in the control group. However, the relative abundance of specific genes as sulfonamide resistance genes was much higher in soil samples supplemented with digestate than without [46]. In a study under lab conditions, Yang et al*.* found an average abundance of ARG of 5.34 × 10^−1^ in food waste compost with 109 different ARG, MGEs and MRGs (out of 153 tested) [85]. Use of food waste compost resulted in the spread of ARGs from fertilizers to soil and to the cultivated pakchoi (also called Chinese cabbage). Bacterial populations from food waste fertilizers significantly altered the bacterial community structure in soil after their application. Transmission happened through different routes, leaf endophyte ARGs came from aerosols. Some ARGs from fertilizers and original soil are transferred from soil to root endophyte and leaf endophyte via a smooth soil-root-endophyte migration pathway (internal pathway). FW compost amended soils showed a higher risk burden of ARGs spreading than FW digestate-amended soils. Biochar combined with FW fertilizers can mitigate the resistome. Consumption of raw vegetables could be a major exposure route for foodborne ARG that is influenced by the presence of ARGs in soil. The authors estimated that the average daily dose of ARGs from raw pakchoi consumption is 10^7^–10^9^ copies/d/kg which could have a potential health risk [85]. More research is needed on the routes of AMR spreading from FW based fertilizers.

#### AMR in leachates from food waste biotreatments

*FW compost.* Composting is often done on a small scale and decentralized manner in rural areas. Small-scale operations might result in suboptimal composting conditions and possible leaching risk. Huang et al*.* collected compost samples from small organic waste treatment stations in rural areas of China [92]. Raw materials of the compost samples included food waste, fruits and vegetables, aquatic products but also chicken and cow manure from rural farming. ARGs and pathogens are measured in solid phase and in the water extract (compost tea). Overall, ARG contamination in rural organic waste composts remains high. The relative abundance of Sulfonamides-ARGs (*-sul1 and sul2)* and tetracyclines-ARGs (*tet-C, tet-X*) in compost samples and water extract was in the same order of magnitude as landfill waste, leachate and sludge, and other environmental pollutants. There was no significant difference in the order of magnitude between the ARG contamination in the compost and its water extract. Therefore, the use of water compost could increase ARG contamination in agricultural fields and may also increase the risk of transmission to runoff and groundwater.

*FW anaerobic digestion.* In addition to the solid digestate, AMR risk might also be associated with liquid digestate or leachates from FW treated by AD. He et al*.,* studied the fate of AMR along AD treatment process and after complementary treatment of leachates in wastewater treatment plant (WWTP). It was conducted in China on an AD plant for FW from restaurants and canteens. In this study, FW anaerobic treatment, the primary treatment facilities mainly involved with physical processes, the later AD system and wastewater treatment in treatment plants (yielding WS and WE) applied biological processes. Most antibiotics and ARGs were recalcitrant during FW treatment processes. But after additional WWTP, nearly all ARGs were transferred to the excess sludge (WS) and, except for *tet-M*, were undetectable in the effluent (WE). Biologically focused WWTP facilities were the most efficient processes in removing ARGs except for quinolones that were enriched. In the effluent (WE) ciprofloxacin was at 2.7 × 10^4^ ± 3.6 × 10^2^ ng/L and ofloxacin at 5.4 × 10^4^ ± 5.1 × 10^2^ ng/L. Those levels were much higher (3–10 times) than other untreated effluents of hospital and livestock farms wastewaters [93]. Interestingly, the antibiotic contamination in FW treatment was primarily caused by human-source antibiotics (81%) (sulfamethoxazole, ciprofloxacin and ofloxacin). Partial least squares analysis revealed that heavy metals influenced antibiotics concentrations variance and most ARGs except *mef-A* and *tet-M* had strong associations with studied MGEs. In this study AD seems to be insufficient to reduce AMR risk but following WWTP removed most of the risk associated with the effluents.

By comparing one and two stage AD, Jang et al*.* evaluated, based on absolute abundance of ARGs in effluent and flow rates of both systems, that the total emission amounts of ARGs in the environment for one-stage and two-stages AD were respectively 4.6 × 10^14^ and 8.3 × 10^11^ copies/day [44].

#### AMR in aerosols from food waste biotreatments

A study showed that aerosols in composting plants harbor abundant and diverse ARGs [94]. The highest concentration of ARGs and MGE *intl1* was detected in the packaging areas. *intl1* had the maximum average concentration of 10^4^ copies/m^3^, with up to (1.7 ± 0.4) × 10^−2^ copies/16S rRNA copy. Aerosolized ARG fragments from composting and packaging areas were also shown to contribute to the compositions of ARG aerosols detected in offices and downwind areas of the composting plant.

Another study showed that ARGs could be found in aerosols during storage of biogas digestate. There was no significant correlation between the relative abundance of ARGs in biogas digestate and aerosols. Storage time (30 days) reduced the total relative abundance of ARGs in digestate but increased the abundance of some ARG subtypes, including *tetM*, *tetX*, *tetQ*, *tetS, ermF* and *sul2* [95].

More data are needed to evaluate the risk associated with aerosols. But optimization of composting and biogas plants like closed area with negative pressure might help to mitigate risk of ARGs dispersion via aerosols.

### Disposal of FW in landfills and open dumps

In most countries, a major part of FW ends up in landfills: up to 60% in China and in the U.S. FW represents an important part of municipal solid waste landfills, from 20 up to 65% of the overall waste found in municipal landfills [96, 97]. FW has high organic content and that could greatly favor microbial growth and potential ARGs transfers [55]. Landfills are important reservoir of antibiotic residues, ARB and ARGs [98]. Data showed that leachate-borne antibiotics could be found at the μg/L level in landfills [51, 99, 100]. Very wide range and relatively high abundance of ARGs are found in landfill, both in leachate and refuse [53–55]. ARG abundances ranged from − 5.5 to − 0.049 log_10_ copies/16 S rRNA or 2.57–10.715 log_10_ copies/g [98]. In addition to the selective pressure of antibiotic residues, the level and the diversity of ARGs is influenced by other factors such as waste type, degradation degree, landfill depth, landfill age and the presence of other chemicals [55, 98].

In landfills, FW could be mixed with multiple chemicals and biological substances from multiple origins: industrial, agricultural and medical. Some are well known to favor selection and HGT of AMR, like disinfectants, various pharmaceuticals, microplastics and heavy metals [51–55]. Based on the literature, Li et al*.* 2024 reported that the heavy metal concentration, ranged from 0.96 to 10,000 mg/kg in refuse 6 × 10^–4^ to 2.43 × 10^1^ mg/L in leachate from landfills. ARB and ARGs could come from FW or other sources and could spread from one type of waste to another [55].

AMR in landfill could be spread to the environment via several routes [99, 101]. The range of ARGs levels detected in refuse and leachate could be similar [55]. However, **leachate** might represent a higher risk of propagation. Dumping sites are often close to highly populated areas and can be above groundwater sources. Landfill leachates carry abundant resistant microorganisms and ARGs (about 10^–5^ to 10^–1^ ARGs copies/16sRNA [55, 102] − 6.301–0.197 log_10_ copies/16 S rDNA [98]). In limited waste management conditions, leachates could lead to AMR contamination of surface and groundwater [51, 53, 54, 103]. Groundwater is of particular concern when used for domestic consumption or agriculture because it is typically consumed with little to no water treatment. There are greater AMR exposure risks around older landfills as they are more prone to leachate and leachate has been found to contain high levels of ARGs [51, 52, 104]. In addition, the proportion of ARG associated to human pathogens, belonging mostly to *Proteobacteria,* increases in older landfills.

An important proportion of municipal solid waste landfills are open dump sites. Food waste in landfills provides accessible food sources for **scavenging animals** like rodents, feral animals but also to migrating birds that could contribute to the spread of AMR. Antimicrobial resistant bacteria including those with significant clinical importance have been identified on animals feeding on landfills [105, 106]. Animals, like landfill foraging gulls, could disperse AMR over long distances [107].

In addition, landfills are a major source of microbial air pollution through airborne microorganisms (*Staphylococcus, Bacillus cereus* and *Pseudomonas aeruginosa*) associated with aerosol/bioaerosols [108, 109]. Landfill-specific airborne particles could be loaded with several types of ARGs. They are effective vectors for the transmission of AMR materials from landfills to the neighboring populations. Close to landfill, the human exposure to ARGs by inhalation could be as high as (5.83 ± 0.16) × 10^5^ copies of ARGs, being ten-fold higher than that ingestion of drinking water [99].

Diverting FW from landfill and valorization of food waste by biotransformation, taking AMR risk into consideration, is by far the preferential strategy for food waste management.

## Conclusions

Antimicrobial residues, ARB and ARGs can be found throughout the food chain, including in FW. It is essential to consider FW within the broader AMR risk landscape and as a vehicle for AMR spread.

Disposal of FW in landfills and open dumps could increase the AMR risk, as it is mixed with other wastes that increase the AMR risk (e.g., heavy metals, microplastics). Routes for AMR spreading are also numerous (leachate, aerosols, wildlife, even human direct interactions) and should be avoided.

In the context of the circular economy, various FW biotreatments are strongly encouraged, as they allow energy and nutrient recovery and can reduce some negative environmental impacts. However, fate of AMR has been poorly considered in the biotreatments of FW, as products resulting from these processes, such as fertilizers, could reintroduce AMR into the food chain. Therefore, a better understanding of AMR risk in FW management processes is essential. While data are available for other types of organic waste, (e.g. such as sludge and manure), they cannot be directly extrapolated to FW. Specific data on different types of FW are strongly needed.

Several studies showed that ARG and ARBs abundance could be reduced by various processes, in particular those involving high temperatures. However, further optimization of these processes is still needed. For example, certain pre-treatments appear to have a great impact on reducing AMR in subsequent treatments (composting or AD). Conversely, some processes are concerning, as they may increase ARGs levels. A lack of controlled processes and AMR surveillance might lead to the dispersion of ARGs into the environment through contaminated compost and digestates applied on cultivated soils, and potentially reintroduce them into the food chain, animals and humans. The insufficient elimination of antibiotics and ARGs in conventional compost and digestate fertilizer poses considerable threat to agriculture safety and human health. There are several limitations in the available data.

- FW sources vary considerably between studies, from using a single type of raw material to mix-food compounds (from both animal and plant origin), and sometimes with additional residues. FW is also influenced by multiple factors ((i.e., geographic origin, culture, climate, season, source and treatment method of waste) [110]. The initial ARGs content can be highly variable. The food composition (e.g., C/N ratio; water content; AMR residues) will impact the efficacy of the biotransformation, the compositions of microbial populations, the initial content and fate of ARGs.
- Biotransformation processes are also highly variable (pre-treatment, mono- or co-composting/AD, volumes, time, parameters, scale) making difficult to generalize results from one process to another. The sources and volumes treated can differ drastically. Furthermore, biotransformation processes are performed at local and individual level, and from small volumes (uncontrolled) up to industrial scale.
- Methods to measure and report ARGs, ARB and AMR residues also vary widely across studies. Studies that conduct direct comparisons using standardized methods are particularly useful as they reduce methodology and/or laboratory bias.
- One of the main unknowns is the extent of AMR spreading from biotransformed FW products into the environment, and potentially, to animals and humans via direct contact, aerosols or through food (when spread as fertilizers on crops). Longitudinal studies measuring AMR in the surrounding environment of biotransformation plants and in food chains are needed.
- More data from low-and middle-income countries (LMICs) is needed. Indeed, LMICs might be at greater risk of AMR and other hazards related to FW. In several countries, AMU is poorly regulated and there is minimal or no surveillance systems to monitor ARB in food products [111]. Both AMU and FW are projected to increase dramatically in LIMCs in the coming years [112, 113]. FW are usually poorly disposed and processed. The incidence of ARB may be higher in LMICs, where humans have often more interaction with animals, waste and the environment, and where infectious disease rates are high.
- Antimicrobial resistance has been mostly studied in bacteria, but there are growing concerns about antifungal resistance. Unlike bacterial infections, the antifungal drug arsenal is limited: it consists mainly on three classes: azoles, echinocandins, and polyenes. There is currently no data available.
- It is difficult to fully assess the level of AMR risk associated with FLW compare to other types of waste. The levels of ARB and ARGs in FW may be comparable to those in animal waste, but show large variability. The total volume of FLW is about a third of the total volume of animal feces generated per year (1.3 billion tons vs. 3.13 billion tons) [114]. However, AMR risk in FLW may be underestimated, potentially leading to less stringent control processes and increased exposure risk.

The circular economy has undeniable social and environmental benefits and should be strongly encouraged. Notwithstanding, ensuring the safety of reuse and recycling processes is essential. It is necessary to improve FW biotransformation processes such as AD and composting, not only to favor yields and efficacy but to will reduce the associated AMR risk as well. Having data on AMR in FLW is essential to have a better surveillance. It could be important to collect those data more globally at country level, in surveillance system as “The International FAO Antimicrobial Resistance Monitoring system”, an IT platform and related FAO activities that assist countries in collecting, analyzing and effectively utilizing their AMR monitoring and surveillance data primarily from livestock, fisheries, and aquaculture, along with their associated food products [115].

The risk of AMR related to FW is a direct consequence of both antimicrobial overuse and excessive generation of FLW. The data presented in this review reinforce the need to reduce both AMU and FLW. AMR in FLW need to be consider in global initiatives as Reduce the Need for Antimicrobials on Farms for Sustainable Agrifood Systems Transformation, a program launched by FAO [116]. It will also help to inform policy on the need to improve AMR surveillance within food waste management systems, and more broadly, underscore the need to integrate FLW into the One Health framework.

## Data Availability

Not applicable.

## Abbreviations

AcoD: Anaerobic co-digestion
AD: Anaerobic digestion
AF: Aerobic fermentation
AM: Antimicrobial
AMR: Antimicrobial resistance
AMU: Antimicrobial use
ARB: Antimicrobial resistant bacteria
ARGs: Antimicrobial resistant genes
BSFL: Black soldier fly larvae
BTC: Batch-fed composting
C/N: Carbon to nitrogen ratio
FAO: Food and Agriculture Organization from the United Nations
FLW: Food loss and waste
FL: Food loss
FW: Food waste
HGT: Horizontal gene transfer
HMs: Heavy metals
LMICs: Low-and-middle income countries
MGEs: Mobile genetic elements
MPs: Microplastics
nZVI: Nanoscale zero valent iron
WWTP: Wastewater treatment plant

## References

[1] Chang Q, Wang W, Regev-Yochay G, Lipsitch M, Hanage WP. Antibiotics in agriculture and the risk to human health: How worried should we be? Evol Appl. 2015;8:240–7. 25861382 10.1111/eva.12185 PMC4380918

[2] Tiedje JM, Fu Y, Mei Z, Schäffer A, Dou Q, Amelung W, et al. Antibiotic resistance genes in food production systems support one health opinions. Curr Opin Environ Sci Heal. 2023;34:100492.

[3] Tiseo K, Huber L, Gilbert M, Robinson TP, Van Boeckel TP. Global trends in antimicrobial use in food animals from 2017 to 2030. Antibiotics. 2020;9:1–14. 10.3390/antibiotics9120918 PMC7766021 33348801

[4] Taylor P, Reeder R. Antibiotic use on crops in low and middle-income countries based on recommendations made by agricultural advisors. CABI Agric Biosci. 2020;1:1–14.

[5] Verhaegen M, Mahillon J, Caulier S, Mingeot‐Leclercq M-P, Bragard C. Data collection on antibiotics for control of plant pathogenic bacteria. EFSA Support Publ. 2024;21:8522E.

[6] Larsson DGJ, Flach CF. Antibiotic resistance in the environment. Nat Rev Microbiol. 2022;20:257–69. 34737424 10.1038/s41579-021-00649-x PMC8567979

[7] Koutsoumanis K, Allende A, Álvarez-Ordóñez A, Bolton D, Bover-Cid S, Chemaly M, et al. Role played by the environment in the emergence and spread of antimicrobial resistance (AMR) through the food chain. EFSA J. 2021;19:e06651. 34178158 10.2903/j.efsa.2021.6651 PMC8210462

[8] Brunn AA, Roustit M, Kadri-Alabi Z, Guardabassi L, Waage J. A meta-analysis to estimate prevalence of resistance to tetracyclines and third generation cephalosporins in Enterobacteriaceae isolated from food crops. Antibiotics. 2022;11:1424. 36290083 10.3390/antibiotics11101424 PMC9598472

[9] Brunn A, Kadri-Alabi Z, Moodley A, Guardabassi L, Taylor P, Mateus A, et al. Characteristics and global occurrence of human pathogens harboring antimicrobial resistance in food crops: a scoping review. Front Sustain Food Syst. 2022;6:824714.

[10] Carvalheira A, Silva J, Teixeira P. Lettuce and fruits as a source of multidrug resistant *Acinetobacter* spp. Food Microbiol. 2017;64:119–25. 28213015 10.1016/j.fm.2016.12.005

[11] Meyer C, Price S, Ercumen A. Do animal husbandry operations contaminate groundwater sources with antimicrobial resistance: systematic review. Environ Sci Pollut Res. 2024;31:16164–76. 10.1007/s11356-024-31899-w PMC10894137 38321277

[12] Ben Y, Hu M, Zhong F, Du E, Li Y, Zhang H, et al. Human daily dietary intakes of antibiotic residues: dominant sources and health risks. Environ Res. 2022;212:113387. 35513060 10.1016/j.envres.2022.113387

[13] Treiber FM, Beranek-Knauer H. Antimicrobial residues in food from animal origin—a review of the literature focusing on products collected in stores and markets worldwide. Antibiotics. 2021;10:534. 34066335 10.3390/antibiotics10050534 PMC8148204

[14] Arsène MMJ, Davares AKL, Viktorovna PI, Andreevna SL, Sarra S, Khelifi I, et al. The public health issue of antibiotic residues in food and feed: causes, consequences, and potential solutions. Vet World. 2022;15:662–71. 35497952 10.14202/vetworld.2022.662-671 PMC9047141

[15] Griboff J, Carrizo JC, Bonansea RI, Valdés ME, Wunderlin DA, Amé MV. Multiantibiotic residues in commercial fish from Argentina. The presence of mixtures of antibiotics in edible fish, a challenge to health risk assessment. Food Chem. 2020;332:127380. 32603916 10.1016/j.foodchem.2020.127380

[16] Founou LL, Founou RC, Essack SY. Antibiotic resistance in the food chain: a developing country-perspective. Front Microbiol. 2016;7:1881. 27933044 10.3389/fmicb.2016.01881 PMC5120092

[17] Tao Q, Wu Q, Zhang Z, Liu J, Tian C, Huang Z, et al. Meta-analysis for the global prevalence of foodborne pathogens exhibiting antibiotic resistance and biofilm formation. Front Microbiol. 2022;13:1783. 10.3389/fmicb.2022.906490 PMC9239547 35774452

[18] EFSA. The European Union summary report on antimicrobial resistance in zoonotic and indicator bacteria from humans, animals and food in 2019–2020. EFSA J. 2022. 10.2903/j.efsa.2022.7209. 10.2903/j.efsa.2022.7209 PMC8961508 35382452

[19] Jans C, Sarno E, Collineau L, Meile L, Stärk KDC, Stephan R. Consumer exposure to antimicrobial resistant bacteria from food at Swiss retail level. Front Microbiol. 2018;9:362. 29559960 10.3389/fmicb.2018.00362 PMC5845543

[20] Himanshu, R. Prudencio C, da Costa AC, Leal E, Chang CM, Pandey RP. Systematic Surveillance and Meta-Analysis of Antimicrobial Resistance and Food Sources from China and the USA. Antibiotics. 2022;11:1471 10.3390/antibiotics11111471 PMC9686904 36358126

[21] Shen Y, Lv Z, Yang L, Liu D, Ou Y, Xu C, et al. Integrated aquaculture contributes to the transfer of mcr-1 between animals and humans via the aquaculture supply chain. Environ Int. 2019;130:104708. 31202027 10.1016/j.envint.2019.03.056

[22] Gunjan, Vidic J, Manzano M, Raj VS, Pandey RP, Chang CM. Comparative meta-analysis of antimicrobial resistance from different food sources along with one health approach in Italy and Thailand. One Heal. 2023;16:100477. 10.1016/j.onehlt.2022.100477 PMC9803827 36593979

[23] Rajaei M, Moosavy MH, Gharajalar SN, Khatibi SA. Antibiotic resistance in the pathogenic foodborne bacteria isolated from raw kebab and hamburger: phenotypic and genotypic study. BMC Microbiol. 2021;21:1-1610.1186/s12866-021-02326–8. 34615465 10.1186/s12866-021-02326-8 PMC8495966

[24] Mdegela RH, Mwakapeje ER, Rubegwa B, Gebeyehu DT, Niyigena S, Msambichaka V, et al. Antimicrobial use, residues, resistance and governance in the food and agriculture sectors, Tanzania. Antibiotics. 2021;10:454. 33923689 10.3390/antibiotics10040454 PMC8073917

[25] Hölzel CS, Tetens JL, Schwaiger K. Unraveling the role of vegetables in spreading antimicrobial-resistant bacteria: a need for quantitative risk assessment. Foodborne Pathog Dis. 2018;15:671–88. 10.1089/fpd.2018.2501. 30444697 10.1089/fpd.2018.2501 PMC6247988

[26] Islam T, Haque MA, Barai HR, Istiaq A, Kim JJ. Antibiotic resistance in plant pathogenic bacteria: recent data and environmental impact of unchecked use and the potential of biocontrol agents as an eco-friendly alternative. Plants. 2024;13(8):1135. 38674544 10.3390/plants13081135 PMC11054394

[27] Datta S, Ishikawa M, Chudhakorn S, Charaslertrangsi T. Prevalence and antimicrobial characteristics of *Escherichia coli* in selected vegetables and herbs in Bangkok, Thailand. J Food Prot. 2024;87:100229. 38246524 10.1016/j.jfp.2024.100229

[28] FAO. Food wastage footprint. Impacts on natural resources. Summary Report. 2013.

[29] FAO. Voluntary code of conduct for food loss and waste reduction. Volunt. Code Conduct food loss waste Reduct. Rome; 2022. 10.4060/cb9433en.

[30] FAO. Food safety in a circular economy [Internet]. Food Saf. a Circ. Econ. Rome: Food Safety and Quality Series, No. 29; 2024; https://openknowledge.fao.org/items/b8e845c9-318d-46fb-acee-a7c6feddd948.

[31] Pearson AJ, Mukherjee K, Fattori V, Lipp M. Opportunities and challenges for global food safety in advancing circular policies and practices in agrifood systems. npj Sci Food. 2024;8:1–8. 39237595 10.1038/s41538-024-00286-7 PMC11377707

[32] Thakali A, MacRae JD. A review of chemical and microbial contamination in food: What are the threats to a circular food system? Environ Res. 2021. 10.1016/j.envres.2020.110635. 33347866 10.1016/j.envres.2020.110635

[33] Zhao C, Xin L, Xu X, Qin Y, Wu W. Dynamics of antibiotics and antibiotic resistance genes in four types of kitchen waste composting processes. J Hazard Mater. 2022. 10.1016/j.jhazmat.2021.127526. 34736188 10.1016/j.jhazmat.2021.127526

[34] Thakali A, MacRae JD, Isenhour C, Blackmer T. Composition and contamination of source separated food waste from different sources and regulatory environments. J Environ Manage. 2022;314:115043. 35429688 10.1016/j.jenvman.2022.115043

[35] Eckstrom K, Barlow JW. Resistome metagenomics from plate to farm: the resistome and microbial composition during food waste feeding and composting on a Vermont poultry farm. PLoS ONE. 2019;14:e0219807. 31751342 10.1371/journal.pone.0219807 PMC6874062

[36] Costa BF, Zarei-Baygi A, Md Iskander S, Smith AL. Antibiotic resistance genes fate during food waste management—comparison between thermal treatment, hyperthermophilic composting, and anaerobic membrane bioreactor. Bioresour Technol. 2023;388:129771. 37739184 10.1016/j.biortech.2023.129771

[37] Sun H, Schnürer A, Müller B, Mößnang B, Lebuhn M, Makarewicz O. Uncovering antimicrobial resistance in three agricultural biogas plants using plant-based substrates. Sci Total Environ. 2022;829:154556. 35306061 10.1016/j.scitotenv.2022.154556

[38] Periasamy J, Krishnamoorthy S, Nagarethinam B, Sivanandham V. Food wastes as a potential hotspot of antibiotic resistance: synergistic expression of multidrug resistance and ESBL genes confer antibiotic resistance to microbial communities. Environ Monit Assess. 2023;195:1–16. 10.1007/s10661-023-11335-1. 10.1007/s10661-023-11335-1 37261634

[39] Yashwant CP, Rajendran V, Krishnamoorthy S, Nagarathinam B, Rawson A, Anandharaj A, et al. Antibiotic resistance profiling and valorization of food waste streams to starter culture biomass and exopolysaccharides through fed-batch fermentations. Food Sci Biotechnol. 2022. 10.1007/s10068-022-01222-9. 37041804 10.1007/s10068-022-01222-9 PMC10082887

[40] Wang P, Qiao Z, Li X, Wu D, Xie B. Fate of integrons, antibiotic resistance genes and associated microbial community in food waste and its large-scale biotreatment systems. Environ Int. 2020;144:106013. 32771831 10.1016/j.envint.2020.106013

[41] Kanger K, Guilford NGH, Lee HW, Nesbø CL, Truu J, Edwards EA. Antibiotic resistome and microbial community structure during anaerobic co-digestion of food waste, paper and cardboard. FEMS Microbiol Ecol. 2020. 10.1093/femsec/fiaa006. 31922542 10.1093/femsec/fiaa006

[42] Liao H, Friman VP, Geisen S, Zhao Q, Cui P, Lu X, et al. Horizontal gene transfer and shifts in linked bacterial community composition are associated with maintenance of antibiotic resistance genes during food waste composting. Sci Total Environ. 2019;660:841–50. 30743970 10.1016/j.scitotenv.2018.12.353

[43] Lin WF, Guo HQ, Zhu LJ, Yang K, Li HZ, Cui L. Temporal variation of antibiotic resistome and pathogens in food waste during short-term storage. J Hazard Mater. 2022;436:129261. 35739780 10.1016/j.jhazmat.2022.129261

[44] Jang HM, Lee J, Shin SG, Shin J, Kim YM. Comparing the fate of antibiotic resistance genes in two full-scale thermophilic anaerobic digestion plants treating food wastewater. Bioresour Technol. 2020;312:123577. 32531733 10.1016/j.biortech.2020.123577

[45] Lee J, Shin SG, Jang HM, Kim YB, Lee J, Kim YM. Characterization of antibiotic resistance genes in representative organic solid wastes: food waste-recycling wastewater, manure, and sewage sludge. Sci Total Environ. 2017;579:1692–8. 27923578 10.1016/j.scitotenv.2016.11.187

[46] Pan X, Yuzuak S, Lou J, Chen L, Lu Y, Zuo JE. Microbial community and antibiotic resistance gene distribution in food waste, anaerobic digestate, and paddy soil. Sci Total Environ. 2023;889:164192. 37196953 10.1016/j.scitotenv.2023.164192

[47] He Y, Yuan Q, Mathieu J, Stadler L, Senehi N, Sun R, et al. Antibiotic resistance genes from livestock waste: occurrence, dissemination, and treatment. npj Clean Water. 2020;3:1–11.

[48] Zhang Y, Cheng D, Xie J, Zhang Y, Wan Y, Zhang Y, et al. Impacts of farmland application of antibiotic-contaminated manures on the occurrence of antibiotic residues and antibiotic resistance genes in soil: a meta-analysis study. Chemosphere. 2022;300:134529. 35395269 10.1016/j.chemosphere.2022.134529

[49] Pean U. Directive 2009/28/EC of the european parliament and of the council of 23 April 2009 on the promotion of the use of energy from renewable sources and amending and subsequently repealing Directives 2001/77/EC and 2003/30/EC. Off J Eur Union. 2009;5:16–62.

[50] Giacometti F, Shirzad-Aski H, Ferreira S. Antimicrobials and food-related stresses as selective factors for antibiotic resistance along the farm to fork continuum. Antibiotics. 2021. 10.3390/antibiotics10060671. 34199740 10.3390/antibiotics10060671 PMC8230312

[51] He P, Huang J, Yu Z, Xu X, Raga R, Lü F. Antibiotic resistance contamination in four Italian municipal solid waste landfills sites spanning 34 years. Chemosphere. 2021;266:129182. 33333336 10.1016/j.chemosphere.2020.129182

[52] Yu Z, He P, Shao L, Zhang H, Lü F. Co-occurrence of mobile genetic elements and antibiotic resistance genes in municipal solid waste landfill leachates: a preliminary insight into the role of landfill age. Water Res. 2016;106:583–92. 27776307 10.1016/j.watres.2016.10.042

[53] You X, Wu D, Wei H, Xie B, Lu J. Fluoroquinolones and Β-lactam antibiotics and antibiotic resistance genes in autumn leachates of seven major municipal solid waste landfills in China. Environ Int. 2018;113:162–9. 29425900 10.1016/j.envint.2018.02.002

[54] Zhang XH, Xu YB, He XL, Huang L, Ling JY, Zheng L, et al. Occurrence of antibiotic resistance genes in landfill leachate treatment plant and its effluent-receiving soil and surface water. Environ Pollut. 2016;218:1255–61. 27593354 10.1016/j.envpol.2016.08.081

[55] Li YJ, Yuan Y, Tan WB, Xi BD, Wang H, Hui KL, et al. Antibiotic resistance genes and heavy metals in landfill: a review. J Hazard Mater. 2024;464:132395. 37976849 10.1016/j.jhazmat.2023.132395

[56] Zhang J, Chen M, Sui Q, Wang R, Tong J, Wei Y. Fate of antibiotic resistance genes and its drivers during anaerobic co-digestion of food waste and sewage sludge based on microwave pretreatment. Bioresour Technol. 2016;217:28–36. 26988135 10.1016/j.biortech.2016.02.140

[57] Ma R, Wang J, Liu Y, Wang G, Yang Y, Liu Y, et al. Dynamics of antibiotic resistance genes and bacterial community during pig manure, kitchen waste, and sewage sludge composting. J Environ Manage. 2023;345:118651. 37499413 10.1016/j.jenvman.2023.118651

[58] Yang X, Sun P, Liu B, Ahmed I, Xie Z, Zhang B. Effect of extending high-temperature duration on ARG rebound in a co-composting process for organic wastes. Sustainability. 2024. 10.3390/su16135284.

[59] Wang H, Lin S, Zhang H, Guo D, Liu D, Zheng X. Batch-fed composting of food waste: microbial diversity characterization and removal of antibiotic resistance genes. Bioresour Technol. 2023;385:129433. 37399965 10.1016/j.biortech.2023.129433

[60] Li X, Wang P, Chu S, Xu Y, Su Y, Wu D, et al. Short-term biodrying achieves compost maturity and significantly reduces antibiotic resistance genes during semi-continuous food waste composting inoculated with mature compost. J Hazard Mater. 2022;427:127915. 34863571 10.1016/j.jhazmat.2021.127915

[61] Ahmed I, Zhang Y, Sun P, Xie Y, Zhang B. Sensitive response mechanism of ARGs and MGEs to initial designed temperature during swine manure and food waste co-composting. Environ Res. 2023;216:114513. 36208781 10.1016/j.envres.2022.114513

[62] Du R, Cui L, Feng Y, Lv X, Gao Y, Li A, et al. Enhancing the decomposition and composting of food waste by in situ directional enzymatic hydrolysis: performance, ARGs removal and engineering application. Waste Manag. 2025;200:114774. 40163955 10.1016/j.wasman.2025.114774

[63] Zhang D, Li X, Li H, Xu Y. Microbial inoculants enhance the persistence of antibiotic resistance genes in aerobic compost of food waste mainly by altering interspecific relationships. Bioresour Technol. 2023;385:129443. 37399957 10.1016/j.biortech.2023.129443

[64] Hou J, Lam KL, Chiu YT, Kwong KY, Lau HL, Marafa LM, et al. Urban green waste bulking agent is the major source of antimicrobial resistance genes persisted in home compost, not animal manure. Environ Res. 2024;242:117713. 38000633 10.1016/j.envres.2023.117713

[65] Han Y, Yang Z, Yin M, Zhang Q, Tian L, Wu H. Exploring product maturation, microbial communities and antibiotic resistance gene abundances during food waste and cattle manure co-composting. Sci Total Environ. 2024;951:175704. 39214357 10.1016/j.scitotenv.2024.175704

[66] Chen Z, Li Y, Ye C, He X, Zhang S. Fate of antibiotics and antibiotic resistance genes during aerobic co-composting of food waste with sewage sludge. Sci Total Environ. 2021;784:146950. 34088024 10.1016/j.scitotenv.2021.146950

[67] Cai M, Dong G, Zhou Y, Yang C, Wu H, Guo C, et al. Product maturation and antibiotic resistance genes enrichment in food waste digestate and Chinese medicinal herbal residues co-composting. Bioresour Technol. 2023;388:129765. 37717706 10.1016/j.biortech.2023.129765

[68] Haffiez N, Chung TH, Zakaria BS, Shahidi M, Mezbahuddin S, Hai FI, et al. A critical review of process parameters influencing the fate of antibiotic resistance genes in the anaerobic digestion of organic waste. Bioresour Technol. 2022;354:127189. 35439559 10.1016/j.biortech.2022.127189

[69] Han Z, Shao B, Lei L, Pang R, Wu D, Tai J, et al. The role of pretreatments in handling antibiotic resistance genes in anaerobic sludge digestion—a review. Sci Total Environ. 2023;869:161799. 36709893 10.1016/j.scitotenv.2023.161799

[70] Cui L, Chen J, Fei Q, Ma Y. The migration regularity and removal mechanism of antibiotic resistance genes during in situ enzymatic hydrolysis and anaerobic digestion of food waste. Bioresour Technol. 2023;385:129388. 37369318 10.1016/j.biortech.2023.129388

[71] Wu Y, Hu W, Huang H, Zheng X, Dong L, Chen Y. Enzymatic pretreatment mitigates the dissemination of antibiotic resistance genes via regulating microbial populations and gene expressions during food waste fermentation. Chin Chem Lett. 2022;34:108058.

[72] Flores-Orozco D, Levin D, Kumar A, Sparling R, Cicek N. A meta-analysis reveals that operational parameters influence levels of antibiotic resistance genes during anaerobic digestion of animal manures. Sci Total Environ. 2022;814:152711. 34974005 10.1016/j.scitotenv.2021.152711

[73] Wang P, Wang X, Chen X, Ren L. Effects of bentonite on antibiotic resistance genes in biogas slurry and residue from thermophilic and mesophilic anaerobic digestion of food waste. Bioresour Technol. 2021;336:125322. 34082336 10.1016/j.biortech.2021.125322

[74] Wang P, Chen X, Liang X, Cheng M, Ren L. Effects of nanoscale zero-valent iron on the performance and the fate of antibiotic resistance genes during thermophilic and mesophilic anaerobic digestion of food waste. Bioresour Technol. 2019;293:122092. 31505392 10.1016/j.biortech.2019.122092

[75] Nghiem LD, Koch K, Bolzonella D, Drewes JE. Full scale co-digestion of wastewater sludge and food waste: bottlenecks and possibilities. Renew Sustain Energy Rev. 2017;72:354–62.

[76] He K, Liu Y, Tian L, He W, Cheng Q. Review in anaerobic digestion of food waste. Heliyon. 2024;10:e28200. 38560199 10.1016/j.heliyon.2024.e28200 PMC10979283

[77] Martín J, Santos JL, Aparicio I, Alonso E. Pharmaceutically active compounds in sludge stabilization treatments: anaerobic and aerobic digestion, wastewater stabilization ponds and composting. Sci Total Environ. 2015;503–504:97–104. 24909712 10.1016/j.scitotenv.2014.05.089

[78] Östman M, Lindberg RH, Fick J, Björn E, Tysklind M. Screening of biocides, metals and antibiotics in Swedish sewage sludge and wastewater. Water Res. 2017;115:318–28. 10.1016/j.watres.2017.03.011. 28288311 10.1016/j.watres.2017.03.011

[79] Wang P, Li X, Chu S, Su Y, Wu D, Xie B. Metatranscriptomic insight into the effects of antibiotic exposure on performance during anaerobic co-digestion of food waste and sludge. J Hazard Mater. 2022;423:127163. 34530275 10.1016/j.jhazmat.2021.127163

[80] Zhang J, Mao F, Loh KC, Gin KYH, Dai Y, Tong YW. Evaluating the effects of activated carbon on methane generation and the fate of antibiotic resistant genes and class I integrons during anaerobic digestion of solid organic wastes. Bioresour Technol. 2018;249:729–36. 29096147 10.1016/j.biortech.2017.10.082

[81] Zhang J, Zhang L, Loh KC, Dai Y, Tong YW. Enhanced anaerobic digestion of food waste by adding activated carbon: fate of bacterial pathogens and antibiotic resistance genes. Biochem Eng J. 2017;128:19–25.

[82] Zhang J, Chua QW, Mao F, Zhang L, He Y, Tong YW, et al. Effects of activated carbon on anaerobic digestion—methanogenic metabolism, mechanisms of antibiotics and antibiotic resistance genes removal. Bioresour Technol Rep. 2019;5:113–20.

[83] Gao P, Gu C, Wei X, Li X, Chen H, Jia H, et al. The role of zero valent iron on the fate of tetracycline resistance genes and class 1 integrons during thermophilic anaerobic co-digestion of waste sludge and kitchen waste. Water Res. 2017;111:92–9. 10.1016/j.watres.2016.12.047. 28061387 10.1016/j.watres.2016.12.047

[84] Li X, Wang P, Chu S, Su Y, Wu D, Xie B. The variation of antibiotic resistance genes and their links with microbial communities during full-scale food waste leachate biotreatment processes. J Hazard Mater. 2021;416:125744. 33862482 10.1016/j.jhazmat.2021.125744

[85] Yang J, Xiang J, Goh SG, Xie Y, Nam OC, Gin KYH, et al. Food waste compost and digestate as novel fertilizers: impacts on antibiotic resistome and potential risks in a soil-vegetable system. Sci Total Environ. 2024;923:171346. 38438039 10.1016/j.scitotenv.2024.171346

[86] Furukawa M, Misawa N, Moore JE. Recycling of domestic food waste: Does food waste composting carry risk from total antimicrobial resistance (AMR)? Br Food J. 2018;120:2710–5.

[87] Mao Y, Akdeniz N, Nguyen TH. Quantification of pathogens and antibiotic resistance genes in backyard and commercial composts. Sci Total Environ. 2021;797:149197. 34311369 10.1016/j.scitotenv.2021.149197

[88] Xie WY, Wang YT, Yuan J, Hong WD, Niu GQ, Zou X, et al. Prevalent and highly mobile antibiotic resistance genes in commercial organic fertilizers. Environ Int. 2022;162:107157. 35219935 10.1016/j.envint.2022.107157

[89] Sun H, Bjerketorp J, Levenfors JJ, Schnürer A. Isolation of antibiotic-resistant bacteria in biogas digestate and their susceptibility to antibiotics. Environ Pollut. 2020;266:115265. 32731190 10.1016/j.envpol.2020.115265

[90] Wolak I, Bajkacz S, Harnisz M, Stando K, Męcik M, Korzeniewska E. Digestate from agricultural biogas plants as a reservoir of antimicrobials and antibiotic resistance genes—implications for the environment. Int J Environ Res Public Health. 2023;20:2672. 36768038 10.3390/ijerph20032672 PMC9915926

[91] Sun H, Levenfors JJ, Brandt C, Schnürer A. Assessing phenotypic and genotypic antibiotic resistance in bacillus-related bacteria isolated from biogas digestates. Ecotoxicol Environ Saf. 2025;291:117859. 39947064 10.1016/j.ecoenv.2025.117859

[92] Huang J, He P, Duan H, Yang Z, Zhang H, Lü F. Leaching risk of antibiotic resistance contamination from organic waste compost in rural areas. Environ Pollut. 2023;320:121108. 36669719 10.1016/j.envpol.2023.121108

[93] He P, Yu Z, Shao L, Zhou Y, Lü F. Fate of antibiotics and antibiotic resistance genes in a full-scale restaurant food waste treatment plant: implications of the roles beyond heavy metals and mobile genetic elements. J Environ Sci (China). 2019;85:17–34. 31471024 10.1016/j.jes.2019.04.004

[94] Gao M, Qiu T, Sun Y, Wang X. The abundance and diversity of antibiotic resistance genes in the atmospheric environment of composting plants. Environ Int. 2018;116:229–38. 29698899 10.1016/j.envint.2018.04.028

[95] Zhang Y, Zheng Y, Zhu Z, Chen Y, Dong H. Dispersion of antibiotic resistance genes (ARGs) from stored swine manure biogas digestate to the atmosphere. Sci Total Environ. 2021;761:144108. 33360136 10.1016/j.scitotenv.2020.144108

[96] Pham TPT, Kaushik R, Parshetti GK, Mahmood R, Balasubramanian R. Food waste-to-energy conversion technologies: current status and future directions. Waste Manag. 2015;38:399–408. 25555663 10.1016/j.wasman.2014.12.004

[97] Ding Y, Zhao J, Liu JW, Zhou J, Cheng L, Zhao J, et al. A review of China’s municipal solid waste (MSW) and comparison with international regions: management and technologies in treatment and resource utilization. J Clean Prod. 2021;293:126144.

[98] Zhang R, Yang S, An Y, Wang Y, Lei Y, Song L. Antibiotics and antibiotic resistance genes in landfills: a review. Sci Total Environ. 2022;806:150647. 34597560 10.1016/j.scitotenv.2021.150647

[99] Wu D, Su Y, Wang P, Zhao J, Xie J, Xie B. Uncover landfilled antimicrobial resistance: a critical review of antibiotics flux, resistome dynamics and risk assessment. Natl Sci Open. 2022;1:20220012.

[100] Anand U, Reddy B, Singh VK, Singh AK, Kesari KK, Tripathi P, et al. Potential environmental and human health risks caused by antibiotic-resistant bacteria (ARB), antibiotic resistance genes (ARGs) and emerging contaminants (ECs) from municipal solid waste (MSW) landfill. Antibiotics. 2021;10:374. 33915892 10.3390/antibiotics10040374 PMC8065726

[101] UNEP. Bracing for Superbugs: Strengthening environmental action in the One Health response to antimicrobial resistance. Geneva; 2023. https://www.unep.org/resources/superbugs/environmental-action.

[102] Wang Y, Wei T, Qiao J, Song L-Y. Occurrence and prevalence of antibiotic resistance in landfill leachate. Environ Sci Pollut Res Int. 2015;22:12525–33. 25903180 10.1007/s11356-015-4514-7

[103] Wang JY, An XL, Huang FY, Su JQ. Antibiotic resistome in a landfill leachate treatment plant and effluent-receiving river. Chemosphere. 2020;242:125207. 31675591 10.1016/j.chemosphere.2019.125207

[104] Wu D, Huang XH, Sun JZ, Graham DW, Xie B. Antibiotic resistance genes and associated microbial community conditions in aging landfill systems. Environ Sci Technol. 2017;51:12859–67. 10.1021/acs.est.7b03797. 28990771 10.1021/acs.est.7b03797

[105] Martín-Maldonado B, Vega S, Mencía-Gutiérrez A, Lorenzo-Rebenaque L, de Frutos C, González F, et al. Urban birds: an important source of antimicrobial resistant Salmonella strains in Central Spain. Comp Immunol Microbiol Infect Dis. 2020;72:101519. 32717528 10.1016/j.cimid.2020.101519

[106] Ahlstrom CA, Bonnedahl J, Woksepp H, Hernandez J, Olsen B, Ramey AM. Acquisition and dissemination of cephalosporin-resistant *E. coli* in migratory birds sampled at an Alaska landfill as inferred through genomic analysis. Sci Rep. 2018;8:7361. 29743625 10.1038/s41598-018-25474-w PMC5943298

[107] Ahlstrom CA, van Toor ML, Woksepp H, Chandler JC, Reed JA, Reeves AB, et al. Evidence for continental-scale dispersal of antimicrobial resistant bacteria by landfill-foraging gulls. Sci Total Environ. 2021;764:144551. 33385653 10.1016/j.scitotenv.2020.144551

[108] Liang Z, Yu Y, Wang X, Liao W, Li G, An T. The exposure risks associated with pathogens and antibiotic resistance genes in bioaerosol from municipal landfill and surrounding area. J Environ Sci (China). 2023;129:90–103. 36804245 10.1016/j.jes.2022.09.038

[109] Breza-Boruta B. The assessment of airborne bacterial and fungal contamination emitted by a municipal landfill site in northern Poland. Atmos Pollut Res. 2016;7:1043–52.

[110] Ortiz-Sanchez M, Inocencio-García PJ, Alzate-Ramírez AF, Alzate CAC. Potential and restrictions of food-waste valorization through fermentation processes. Fermentation. 2023;9:274.

[111] Odey TOJ, Tanimowo WO, Afolabi KO, Jahid IK, Reuben RC. Antimicrobial use and resistance in food animal production: food safety and associated concerns in Sub-Saharan Africa. Int Microbiol. 2024. 10.1007/s10123-023-00462-x. 38055165 10.1007/s10123-023-00462-x PMC10830768

[112] Lopez Barrera E, Hertel T. Global food waste across the income spectrum: implications for food prices, production and resource use. Food Policy. 2021;98:101874.

[113] Van Boeckel TP, Brower C, Gilbert M, Grenfell BT, Levin SA, Robinson TP, et al. Global trends in antimicrobial use in food animals. Proc Natl Acad Sci USA. 2015;112:5649–54. 25792457 10.1073/pnas.1503141112 PMC4426470

[114] Berendes DM, Yang PJ, Lai A, Hu D, Brown J. Estimation of global recoverable human and animal faecal biomass. Nat Sustain. 2018;1:679–85. 38464867 10.1038/s41893-018-0167-0 PMC10922008

[115] FAO. The International FAO Antimicrobial Resistance Monitoring (InFARM) system. Int. FAO Antimicrob. Resist. Monit. Syst. FAO; 2024.

[116] FAO. Reduce the Need for Antimicrobials on Farms for Sustainable Agrifood Systems Transformation. FAO; 2024. https://openknowledge.fao.org/handle/20.500.14283/cd1715en.

